# Cystic glioblastoma: A systematic review and meta-analysis of characteristics and outcomes

**DOI:** 10.1016/j.bas.2022.101692

**Published:** 2022-11-21

**Authors:** Morrakot Sae-Huang, Luke Christopher Smith, Inga Usher, Ciaran Scott Hill

**Affiliations:** a Department of Neurosurgery, The National Hospital for Neurology and Neurosurgery, Queen Square, London, WC1N 3BG, UK; b Department of Neurosurgery, John Radcliffe Hospital, Headington, Oxford, OX3 9DU, UK; c Cancer Biology Division, The UCL Cancer Institute, University College London, London, WC1E 6DD, UK

**Keywords:** Glioblastoma multiforme, Cystic glioblastoma, Characteristics, Prognosis, Systematic review, Meta-analysis

## Abstract

**Introduction:**

Cystic glioblastoma is a well-recognised clinical entity but the characteristics and role of these cystic components in determining clinical outcome remains poorly understood.

**Research question:**

To determine whether (1) there is a prognostic significance to a glioblastoma having a cystic component and (2) whether the presence of cyst, and its prognosis relative to non-cystic glioblastoma, relates to patient demographics and other tumour characteristics.

**Material & methods:**

A systematic review and meta-analysis was conducted in accordance to PRISMA guidelines. Articles with histological and/or radiological diagnosis of cystic glioblastoma that reported on survival outcome and/or characteristics of cystic glioblastomas mentioned were included. Meta-analysis was performed and presented using random effect model.

**Results:**

Twenty studies met the inclusion criteria, and nine studies were included in the meta-analysis (374 glioblastoma patients with cystic components and 2477 glioblastoma patients without cystic components above 18 years of age). Search result did not yield any Level I evidence. There is statistically significant survival benefit in cystic over non-cystic glioblastomas (HR ​= ​0.81, 95%CI 0.70–0.93, p ​= ​0.004, I^2^ ​= ​50%). Studies reported younger average patient age, larger tumor size and slower tumor growth velocity in cystic glioblastoma. No significant difference in gender ratio and IDH-1 and MGMT methylation status between cystic and non-cystic glioblastoma were reported.

**Discussion & conclusion:**

Presence of cyst in glioblastoma tumor is associated with improved overall survival outcome. Etiology of cystic entities and why they might confer survival benefits remained to be determined, and future studies examining how to best treat cystic glioblastomas would be clinically valuable.

## Highlights

• Presence of cyst in glioblastoma tumour is associated with improved overall survival.
• Patients with cystic tumour are younger.
• Cystic tumors have larger size and slower tumour growth velocity.

## 1 Introduction

Glioblastomas are highly aggressive intrinsic brain tumors, comprising a third of central nervous system tumors with an incidence of 3 cases per 100,000 individuals (Ostrom et al., 2019). They display cellular and genetic heterogeneity and have a poor prognosis with rapidly fatal progression in almost all cases (Sharma and Deb, 2011). Since 2005 the standard of care (SOC) is maximal safe resection with adjuvant temozolomide and radiotherapy (Stupp et al., 2005). Outcomes remain poor with a 5-year survival rate of only 5–10% (Stupp et al., 2009). Survival is influenced by patient-specific factors such as age and performance status, and tumor-related factors including anatomical location, size of enhancing tumor, and molecular characteristics such as isocitrate dehydrogenase mutation (IDH) and methylation of the O (Maldaun et al., 2004)-methylguanine-DNA methyltransferase promoter (pMGMT) (Walid, 2008). The presence of cysts within or associated with the tumor has been proposed as a further determinant of clinical outcome. Cystic components are reported in 7–23% of glioblastomas, either as small cysts within large areas of necrosis, or as large cysts comprising more than half of total tumor volume (Maldaun et al., 2004; Utsuki et al., 2006; Choi et al., 2010). While cystic glioblastoma is a well-recognized clinical entity, it lacks a formal radiological, histological or genetic definition. The 2021 World Health Organization (WHO) classification of tumors of central nervous system (CNS) distinguished IDH-wildtype glioblastoma from IDH-mutant astrocytoma; however, cystic glioblastoma is not captured in this classification system or other commonly described molecular profiles (WHO Classification of Tumour Editorial Board, 2021). Instead, cystic gliomas is generally considered an anatomical description recognized on magnetic resonance imaging (MRI) and their prognostic significance is uncertain (Curtin et al., 2020).

Current clinical guidelines do not take into consideration the presence of a cyst in formulation of treatment decisions or prognostication (Curtin et al., 2020). Understanding the significance of cystic components may influence clinical decisions. Several retrospective studies have compared cystic and non-cystic glioblastoma, their clinical associations (age, tumor volume) and survival outcomes. However, there is no consensus regarding the significance of cystic glioblastoma across the existing literature and many previous studies are hampered by small numbers of patients and time courses predating or spanning the current SOC.

The aim of this systematic review and meta-analysis was to study tumor characteristics and survival outcome in adult patients (>18 years old) with histological and/or radiological diagnosis of cystic glioblastoma-either IDH-wildtype glioblastoma or IDH-mutant grade IV astrocytoma (previously termed IDH-mutant glioblastoma-in comparison to those with glioblastomas that do not have a diagnosis of cyst or cystic components. Specifically, the following questions were addressed:

• Is cystic glioblastoma associated with differences in overall survival (OS) compared to non-cystic glioblastoma?
• Is cystic glioblastoma associated with:
  o Age
  o Sex
  o Anatomical location
  o Pre-operative Karnofsky performance status (KPS)
  o Tumor size/volume and growth velocity
  o IDH1/2 mutation and pMGMT methylation
  o Differences in overall survival before and after the SOC (2005)?

## 2 Methods

This systematic review and meta-analysis was conducted according to the Preferred Reporting Items for Systematic Reviews and Meta-Analyses (PRISMA) guidelines (Page et al., 2021). The population, intervention, comparator, outcome (PICO) elements included number of patients, average age, gender, type of intervention, survival outcome including median overall survival for cystic versus non-cystic glioblastoma and hazard ratio (HR) - either reported by the studies or estimated, and characteristics associated with cystic glioblastoma which were median pre-operative KPS, tumor size, tumor growth velocity, molecular markers (IDH1/2 mutation and pMGMT methylation) and anatomical location. Primary summary measures were OS and clinical and patient characteristics associated with cystic glioblastoma.

### 2.1 Definition of cystic glioblastoma

Given the heterogeneity of definition of cystic glioblastoma, the authors' own definition of cystic glioblastoma in the reviewed papers was taken. In some papers cystic glioblastoma was defined as the presence of any cystic component (Choi et al., 2010; Curtin et al., 2020; Li et al., 2016; Pope et al., 2005; Yamasaki et al., 2019) and in others it was a cystic component occupying at least 50% of the tumor volume (Maldaun et al., 2004; Utsuki et al., 2006; Kaur et al., 2011; Sarmiento et al., 2014).

### 2.2 Search strategy

The following search terms were run on eight databases (PubMed, MEDLINE, HMIC, Embase, Emcare, CINALH, BNI and AMED) via National Institute for Health and Care Excellence (NICE) Health Databases Advanced Search (HDAS) on November 10, 2021: (Glioblastoma OR Glioblastoma multiforme OR GBM OR Grade IV astrocytoma OR High-grade astrocytoma) AND (Cyst OR Cystic) using HDAS ‘combine’ function for Boolean operators.

### 2.3 Screening strategy

Eligible studies included randomized controlled trials, observational studies, and retrospective case reviews or case series with three or more patients. Inclusion criteria were (1) patients aged 18 years or above, with (2) histological and/or radiological diagnosis of cystic glioblastoma, and (3) either a report on survival measure (median survival time and/or hazard ratio) and/or clinical characteristics of glioblastoma tumors including anatomical location, pre-operative Karnofsky performance status (KPS), tumor size/volume, tumor growth velocity, IDH 1/2 mutation status and pMGMT methylation. Studies were excluded if they (Ostrom et al., 2019) did not include patients with cystic glioblastoma (Sharma and Deb, 2011), did not provide data on survival or clinical characteristics of cystic glioblastoma (Stupp et al., 2005), reported preclinical data only (Stupp et al., 2009), not in English with no translation available (Walid, 2008), only used previously reported data (Maldaun et al., 2004), are unpublished abstracts. Two authors (MSH, LCS) independently performed the search and reviewed the papers. Conflicting findings were discussed with senior authors to achieve consensus. Duplicates were removed. Article titles and abstracts were reviewed in the screening step, followed by assessment of the full text for final selection.

### 2.4 Meta-analysis

Studies that provided time-to-event OS/or HR were included. For studies that did not report the HR it was estimated using methods described by Parmar et al. (1998). Review Manager, 2020 (RevMan 5.4) (Review Manager, 2020) was used for data analysis. The random effects model was used as opposed to the fixed effect model although both were assessed. Heterogeneity was measured using the I^2^ statistic according to the Cochrane Handbook for Systematic Reviews of Interventions (Higgins et al., 2021).

### 2.5 Subgroup analyses

Subgroup analysis was performed for studies with patients before and after 2005. Due to heterogeneity in the definition of cystic glioblastoma, planned a priori subgroup analysis was performed for studies that described cystic volume of >50% of total tumor volume versus those that considered described any evidence of cystic components.

### 2.6 Risk of bias analyses

Risk of bias analysis was performed using the National Heart, Lung and Blood Institute quality assessment tool for systematic review and meta-analysis (National Heart and Lung and Blood Institute, 2021).

## 3 Results

### 3.1 Literature search

**Fig. 1:**
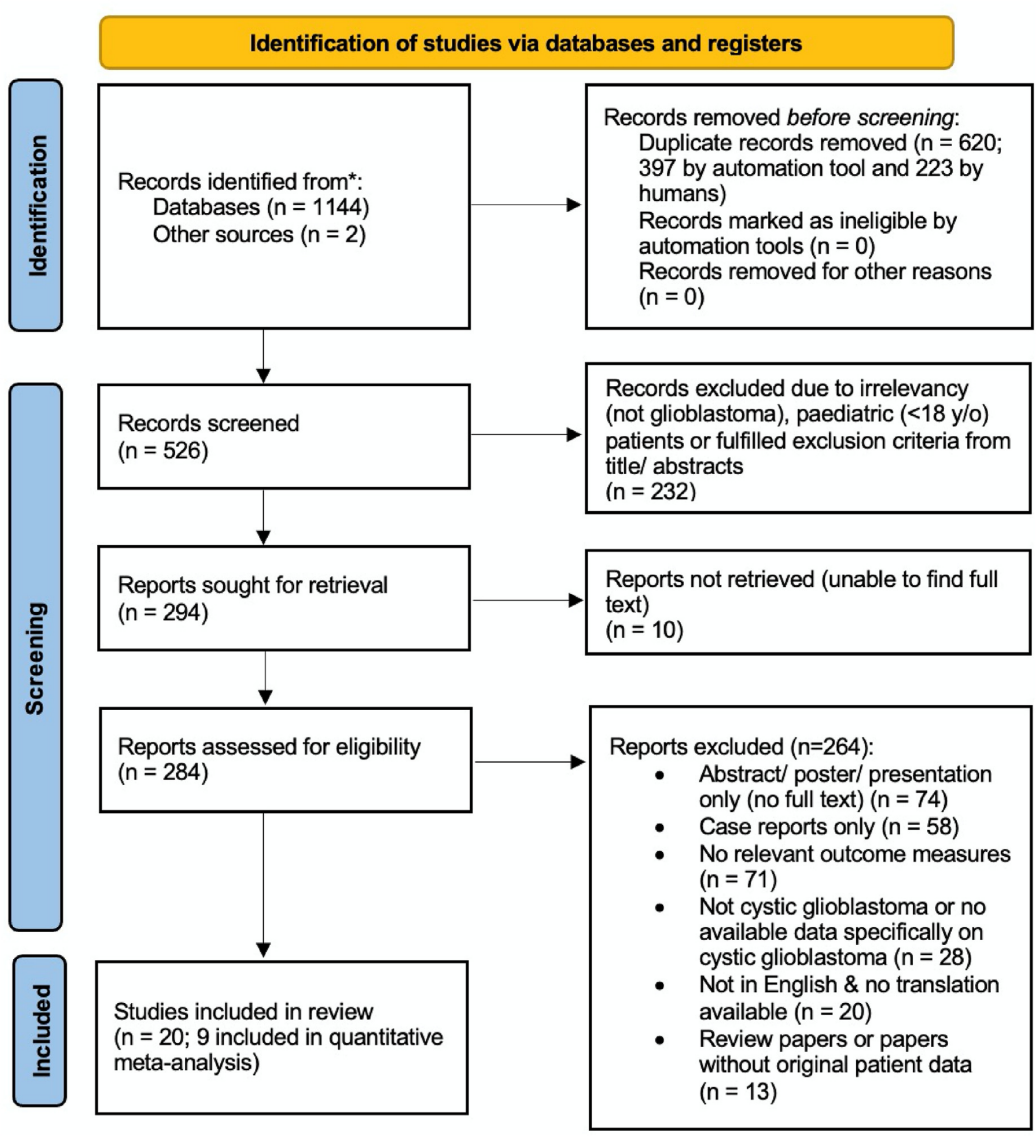
PRISMA flow diagram (Page et al., 2021).

Results from each stage of the literature review are summarized in Fig. 1. After applying our inclusion criteria, 20 studies were included in our systematic review. 17 were retrospective cohort studies and three were case series (Fig. 1). Our search did not yield any Level I evidence.

Twelve studies compared OS in cystic versus non-cystic glioblastoma patients (Table 1). Three studies reported survival without providing quantitative data and were excluded from our meta-analysis. The other nine studies reported the median overall survival time of patients with cystic versus non-cystic glioblastoma. Four of these reported the HR while five provided Kaplan-Meier curves and/or survival rate table which allowed for HR estimation. Therefore, nine studies were included in our meta-analysis.

**Table 1:** Retrospective cohort studies comparing survival outcome of cystic glioblastoma versus non-cystic glioblastoma.

**STUDY**	**PATIENT (n)**		**PATIENT DATA FROM PRE-2005 OR POST-2005 OR BOTH?**	**INTERVENTION**	**OUTCOME DESCRIPTION**	**OVERALL MEDIAN SURVIVAL TIME IN MONTHS (95% CI)**			**HAZARD RATIO**	
						**Cystic**	**Non-Cystic**	**p-value**	**HR (95% CI)**	**p-value**
Choi et al., 2010 (Choi et al., 2010)^b^	Cystic	7	Both	Surgical resection both pre-2005 and current standard of care	Positive overall survival benefit in cystic over non-cystic glioblastoma.	43.8 (N/A)	12.5 (N/A)	0.003	0.80 (0.46–1.39)^a^	0.437^a^
	Non-cystic	14								
Choi et al., 2020 (Choi et al., 2020)^c^	Cystic	40	Post-2005	Current standard of care	Positive overall survival benefit in cystic over non-cystic glioblastoma.	N/A	N/A	N/A	N/A	N/A
	Non-cystic	144								
Curtin et al., 2020 (Curtin et al., 2020)^b^	Cystic	88	Both	Surgical resection both pre-2005 and current standard of care	Positive overall survival benefit in cystic over non-cystic glioblastoma.	22.7 (N/A)	15.2 (N/A)	0.0012	0.77 (0.65–0.92)	0.046
	Non-cystic	405								
Kaur et al., 2011 (Kaur et al., 2011)^b^	Cystic	37	Post-2005	Current standard of care	No significant difference in overall survival outcome.	17 (12.6–21.3)	15.9 (14.6–17.2)	0.99	0.83 (0.56–1.22)	N/A
	Non-cystic	317								
Li et al., 2016 (Li et al., 2016)^b^	Cystic	118	Both	Surgical resection both pre-2005 and current standard of care	Positive overall survival benefit in cystic over non-cystic glioblastoma.	20.3 (15.3–25.5)	13.0 (12.2–13.7)	<0.001	0.60 (0.48–0.75)	<0.001
	Non-cystic	1109								
Maldaun et al., 2004 (Maldaun et al., 2004)^b^	Cystic	22	Pre-2005	Pre-2005 surgical resection	No significant difference in overall survival outcome.	18.2 (11.9–24.5)	14.3 (12.1–16.4)	0.12	0.66 (0.51–0.86)^a^	0.002^a^
	Non-cystic	22								
Sarmiento et al., 2014 (Sarmiento et al., 2014)^b^	Cystic	27	Both	Surgical resection both pre-2005 and current standard of care	No significant difference in overall survival outcome.	15.0 (6.1–30.8)	18.2 (15.6–20.1)	0.77	1.03 (7.78–1.36)^a^	0.831^a^
	Non-cystic	324								
Pope et al., 2005 (Pope et al., 2005)^b^	Total 110 patients^e^		Pre-2005	Pre-2005 surgical resection	No significant difference in overall survival outcome.	N/A	N/A	N/A	0.92 (0.46–1.80)	0.799
Utsuki et al., 2006 (Utsuki et al., 2006)^b^	Cystic	5	Post-2005	Pre-2005 surgical resection	Positive overall survival benefit in cystic over non-cystic glioblastoma.	19.8 (6.7–37.0)	12.8 (2.1–74.2)	<0.05	0.76 (0.57–1.02)^a^	0.064^a^
	Non-cystic	32								
Wu et al., 2015 (Wu et al., 2015)	Cystic	20	Post-2005	Current standard of care	No significant difference in overall survival outcome.	13.03 (6.9–19.2)	14.5 (12.7–16.3)	0.73	N/A	N/A
	Non-cystic	67								
Yamasaki et al., 2019 (Yamasaki et al., 2019)^b^, ^d^	Cystic	10	Post-2005	Bevacizumab in all patients of recurrent glioblastoma	No significant difference in overall survival outcome.	N/A	N/A	0.99	1.14 (0.87–1.50)^a^	0.351^a^
	Non-cystic	73								
Zhou et al., 2018 (Zhou et al., 2018) f	Total 110 patients^e^		Both	Surgical resection both pre-2005 and current standard of care	Positive overall survival benefit in cystic over non-cystic glioblastoma.	N/A	N/A	0.045	N/A	N/A

N/A indicates that relevant quantitative data were not presented in the paper.

a HR and 95% CI estimated from either Kaplan-Meier curve or rate of survival table provided in the studies.

b Studies included in the meta-analysis.

c No quantitative data presented in the paper.

d Study described recurrence cases.

e Paper did not indicate specific number of cystic versus non-cystic number of patients.

f Study includes patient under the age 18.

One additional study comparing the rate of cystic changes in long-term versus short-term survivors in patients with IDH-wildtype and mutant glioblastoma was included (Jiang et al., 2021).

Meta-Analysis for Primary Endpoint: Cystic glioblastoma is associated with a positive overall survival benefit.

**Fig. 2:**
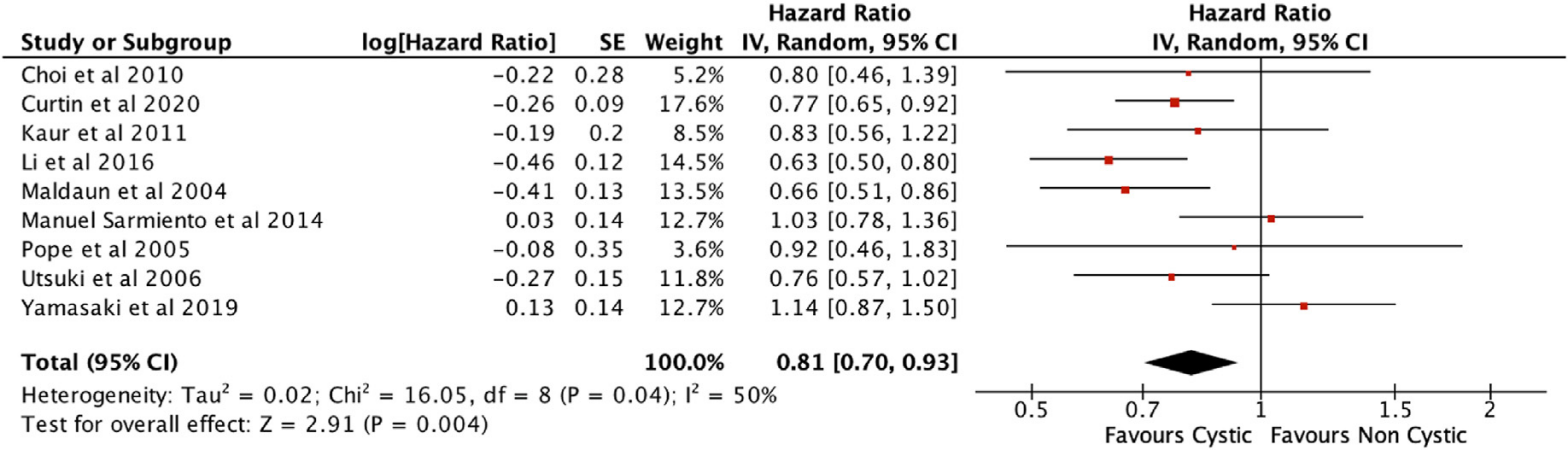
Forest plot of meta-analysis.

Random effects meta-analysis of the nine studies in Table 1 revealed a survival benefit in cystic over non-cystic glioblastomas, with moderate heterogeneity between studies (HR ​= ​0.81, 95%CI 0.70–0.93, p ​= ​0.004, I^2^ ​= ​50%) (Fig. 2).

A total of 3141 patients from 12 studies were included in our review comparing the impact on survival of cystic versus non-cystic glioblastoma. We included 374 patients with cystic glioblastoma and 2477 patients with non-cystic glioblastoma as defined by the authors of each study. We also included 290 patients from studies where the relative numbers of cystic and non-cystic cases were not specified (Pope et al., 2005; Zhou et al., 2018) (Table 1).

Of the three studies that were not included in the meta-analysis due to lack of quantitative data on HR or median survival, two suggested a positive survival outcome in cystic glioblastomas (Zhou et al., 2018; Choi et al., 2020) while the other reported no difference in survival (Wu et al., 2015).

In addition, one study compared the rate of cystic components in long-term survivors versus short-term survivors, defined as patients who survived beyond five years (n ​= ​45) and less than five years (n ​= ​167) after diagnosis. They demonstrated that long-term survivors demonstrated a higher frequency of cystic change compared to short-term survivors (56.8% *vs.* 19.2%, *P ​<* 0.001) (Jiang et al., 2021), and that cystic change is a predictive factor for long-term survivors (OR ​= ​3.791, 95% CI: 1.082–13.275, *P ​=* 0.037) (Jiang et al., 2021).

### 3.2 Subgroup analysis I: stratification based on standard of care

**Fig. 3:**
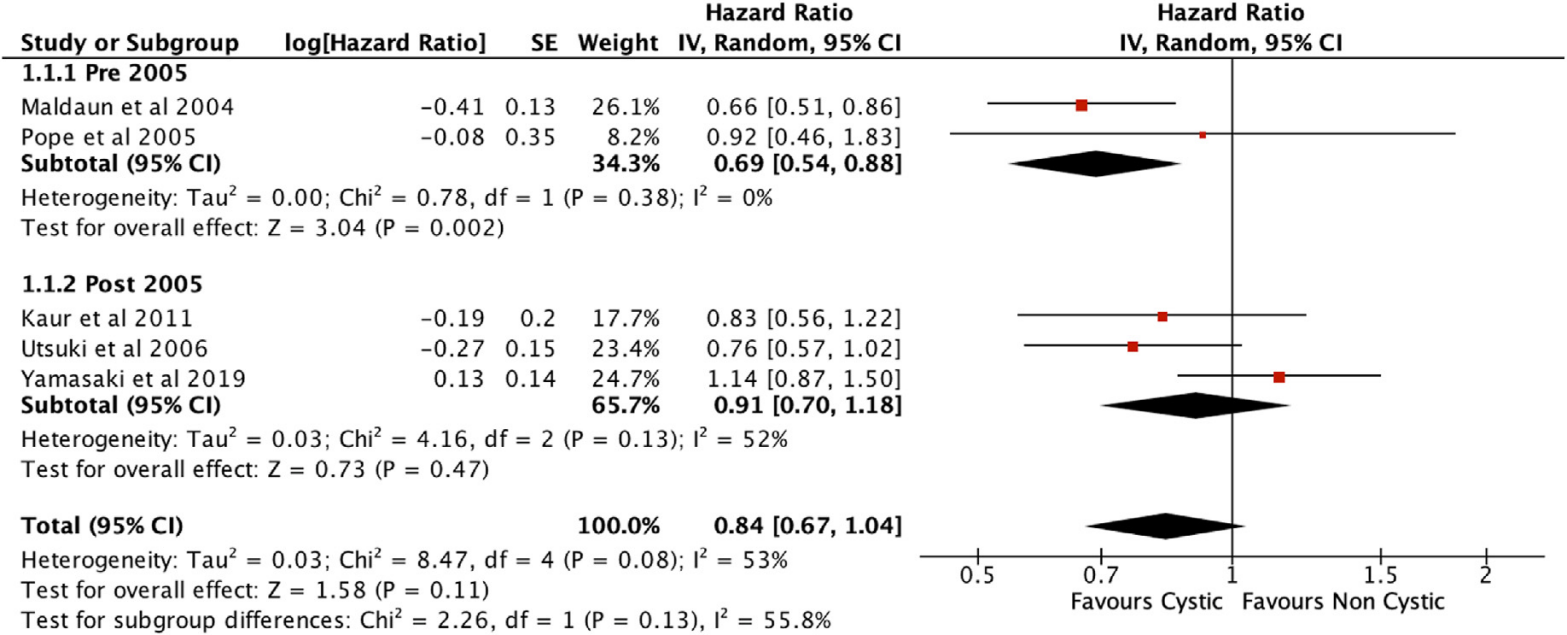
Subgroup analysis I: Pre-2005 vs Post-2005, excluding studies with both.

As the current standard of care was established in 2005 (Stupp et al., 2005), patients pre- and post-2005 may have been treated differently and this may be a potential confounding factor. Studies which included patients treated pre-2005 only and studies with patient samples post- 2005 only were compared. Studies spanning both pre- and post-2005 patients were excluded from this subgroup analysis. There was no difference in overall survival between these two subgroups (Chi^2^ ​= ​1.21, df ​= ​1 (P ​= ​0.27), I^2^ ​= ​17.3%) (Fig. 3).

Four papers included patients treated both before and after 2005 and did not analyze the survival outcomes of the two groups separately (Choi et al., 2010; Li et al., 2016; Sarmiento et al., 2014; Zhou et al., 2018). Curtin et al. (2020) reported that although patients with cystic glioblastoma (n ​= ​88) survived for significantly longer compared to their entire cohort who were treated before and after 2005 (n ​= ​493, p ​= ​0.012), they found no significant difference when comparing cystic versus non-cystic cases who received the SOC after 2005 only (n ​= ​184, p ​= ​0.29). Similarly, among the six papers (Maldaun et al., 2004; Utsuki et al., 2006; Choi et al., 2010, 2020; Li et al., 2016; Zhou et al., 2018) that described a survival benefit in cystic over non-cystic glioblastoma, four studies included patients treated before 2005 (Choi et al., 2010; Curtin et al., 2020; Li et al., 2016; Zhou et al., 2018). However, the subgroup analysis of studies with patient data from post-2005 only (n ​= ​474) (Utsuki et al., 2006; Yamasaki et al., 2019; Kaur et al., 2011) versus those with patient data from pre-2005 only (n ​= ​154) (Maldaun et al., 2004; Pope et al., 2005) did not show a significant difference between the two subgroups (p ​= ​0.13).

Furthermore, Curtin (Curtin et al., 2020) reported that patients with cystic glioblastoma did not appear to benefit from the current SOC (no statistical difference between pre- and post-2005, p ​= ​0.28), but those with non-cystic glioblastoma showed the expected overall survival benefit with the SOC (p ​< ​0.0001). The study also showed that patients with IDH1 wildtype cystic glioblastoma did not show a survival benefit from current SOC (p ​= ​0.99) while those with non-cystic IDH1 wildtype showed a positive survival benefit (p ​< ​0.0001).

### 3.3 Subgroup analysis II: stratification based on heterogenous definition of cystic glioblastoma

**Fig. 4:**
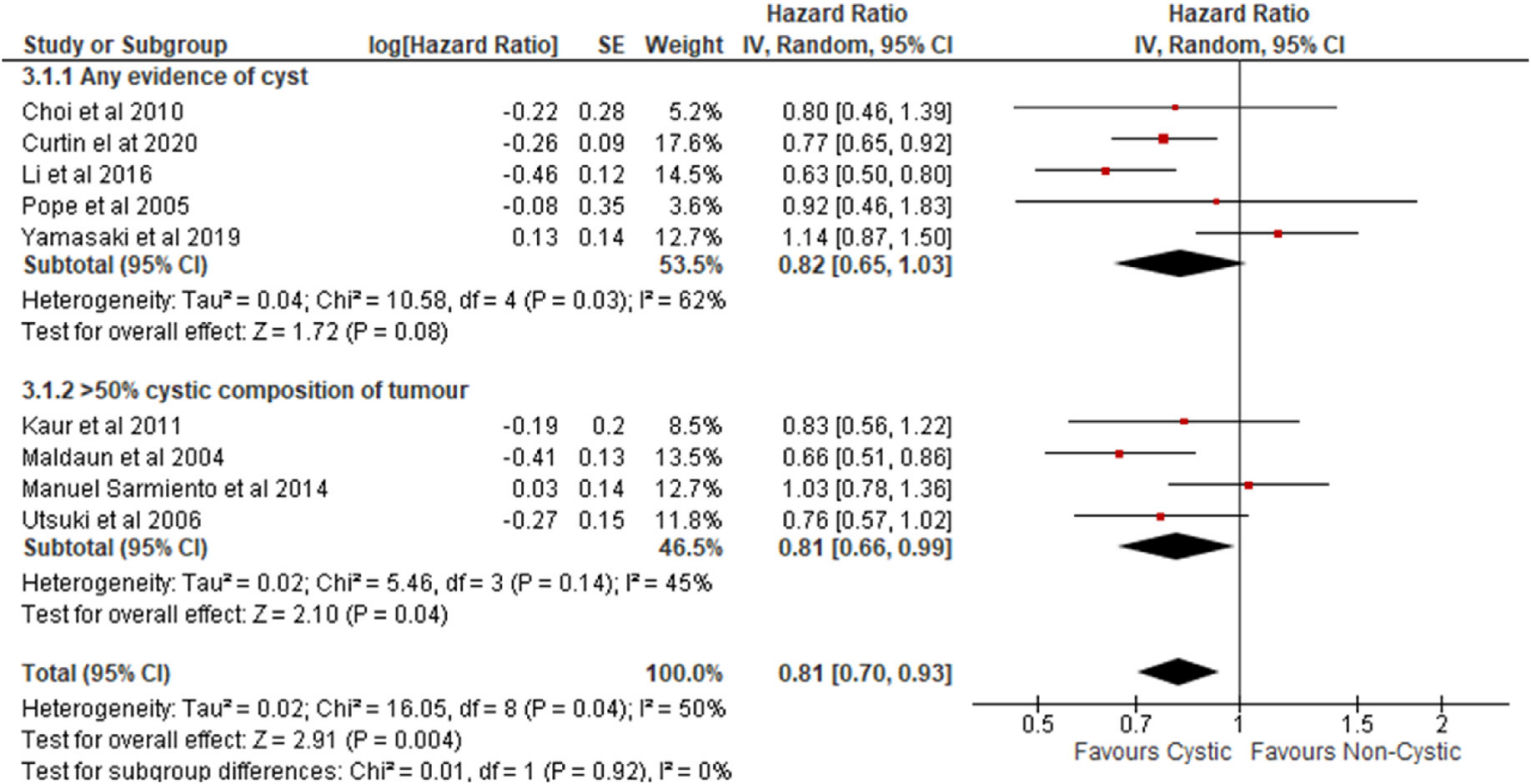
*Subgroup analysis II: Any evidence of cyst versus >50% cystic composition of tumor*.

Outcomes in studies that included any evidence of a tumor cyst (Choi et al., 2010) (Curtin et al., 2020; Li et al., 2016; Pope et al., 2005; Yamasaki et al., 2019) were compared with those that only considered ‘cystic’ tumors as those that occupied more than 50% of the tumor volume (Maldaun et al., 2004; Utsuki et al., 2006; Kaur et al., 2011; Sarmiento et al., 2014) in subgroup analyses. No significant difference in overall survival was identified between these subgroups (Chi^2^ ​= ​0.01, df ​= ​1, (P ​= ​0.92), I^2^ ​= ​0%) (Fig. 4).

### 3.4 Risk of bias analysis: studies included in the review are at a high risk of bias

**Fig. 5:**
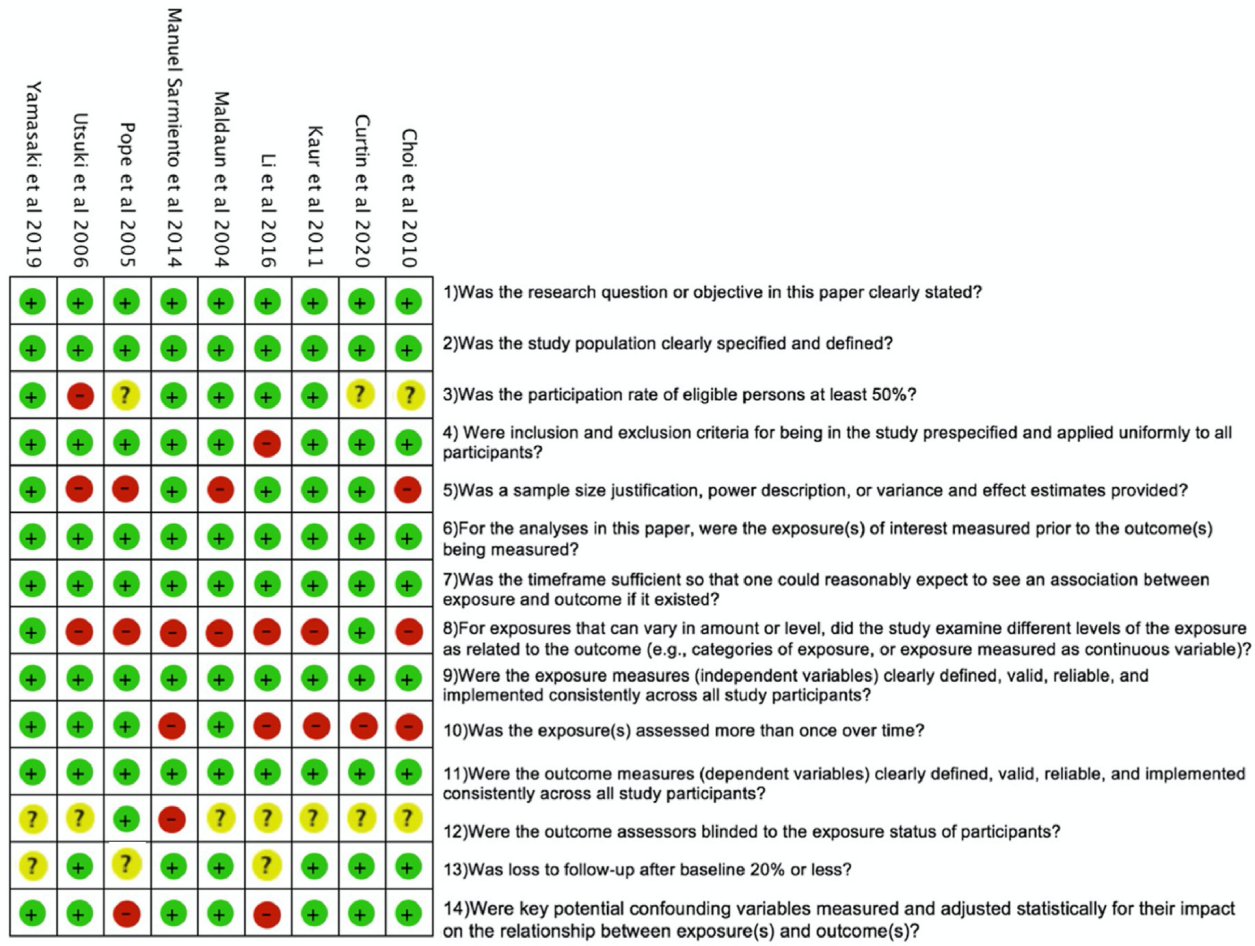
Risk of bias table (National Heart and Lung and Blood Institute, 2021).

All studies were assessed to be at high risk of bias rendering a sensitivity analysis that removes studies at high risk of bias impractical (Fig. 5) (National Heart and Lung and Blood Institute, 2021).

### 3.5 Patients with cystic glioblastomas are younger

Seven studies compared the characteristics of patients with cystic versus non-cystic glioblastoma, specifically sex, age, median pre-operative KPS, mean tumor size on pre-operative imaging and molecular markers (IDH1 mutation and pMGMT methylation status) (Table 2). Six of these seven studies (all except Choi et al.‘s study (Choi et al., 2020)) were included in the meta-analysis.

**Table 2:** Retrospective cohort studies comparing patient demographics, pre-operative KPS, mean tumor size, tumor growth velocity and molecular markers of cystic GBM vs non-cystic GBM.

STUDY	PATIENT (n)		GENDER (% MALE)		MEAN AGE AND RANGE (YEARS)		MEDIAN PRE-OP KPS		MEAN TUMOUR SIZE ON PRE-OP IMAGING		TUMOUR GROWTH VELOCITY MEASURED BY T1Gd (mm/day)		MOLECULAR MARKERS (%)	
Choi et al., 2020 (Choi et al., 2020)	Cystic	23	Cystic	52.2%	Cystic	55.5	Cystic	N/A	Cystic	N/A	Cystic	N/A	IDH-1 mut	
	Non-cystic	119	Non-cystic	54.9%	Non-cystic	52.3	Non-cystic	N/A	Non-cystic	N/A	Non-cystic	N/A	Cystic	10.5%
			p-value	0.926	p-value	0.277	p-value	N/A	p-value	N/A	p-value	N/A	Non-cystic	3.4%
													p-value	0.314
													
													pMGMT met	
													Cystic	59.1%
													Non-cystic	48.0%
													p-value	0.223
Curtin et al., 2020 (Curtin et al., 2020)	Cystic	88	Cystic	53.4%	Cystic	53.3	Cystic	90 (N/A)	Cystic	51020 ​mm^3^^b^	Cystic	0.03024	IDH-1 mut	
	Non-cystic	405	Non-cystic	63.2%	Non-cystic	59.1	Non-cystic	80 (N/A)	Non-cystic	31140 ​mm^3^^b^	Non-cystic	0.1149	Cystic	84%
			p-value	0.087	p-value	<0.05	p-value	0.021	p-value	<0.0001	p-value	0.039	Non-cystic	92%
													p-value	0.19
													
													pMGMT met	
													Cystic	41%
													Non-cystic	31%
													p-value	0.40
Kaur et al., 2011 (Kaur et al., 2011)	Cystic	37	Cystic	56.8%	Cystic	54 (21–83) a	Cystic	90 (30–90)	Cystic	5.3 ​cm	Cystic	N/A	IDH-1 mut	
	Non-cystic	217	Non-cystic	60.0%	Non-cystic	58 (24–88) a	Non-cystic	90 (60–100)	Non-cystic	4.6 ​cm	Non-cystic	N/A	Cystic	N/A
			p-value	0.79	p-value	0.05	p-value	0.06	p-value	<0.01	p-value	N/A	Non-cystic	N/A
													p-value	N/A
													
													pMGMT met	
													Cystic	N/A
													Non-cystic	N/A
													p-value	N/A
Sarmiento et al., 2014 (Sarmiento et al., 2014)	Cystic	27	Cystic	55.6%	Cystic	55.4	Cystic	80 (70–90)	Cystic	28% (>6 ​cm)	Cystic	N/A	IDH-1 mut	
	Non-cystic	324	Non-cystic	61.4%	Non-cystic	60.6	Non-cystic	80 (70–90)	Non-cystic	28% (>6 ​cm)	Non-cystic	N/A	Cystic	7.4%
			p-value	0.16	p-value	0.16	p-value	0.24	p-value	0.63	p-value	N/A	Non-cystic	N/A
													p-value	N/A
													
													pMGMT met	
													Cystic	N/A
													Non-cystic	N/A
													p-value	N/A
Utsuki et al., 2006 (Utsuki et al., 2006)	Cystic	5	Cystic	40%	Cystic	44 (26–59) a	Cystic	80 (70–90)	Cystic	80% (>5 ​cm)	Cystic	N/A	IDH-1 mut	
	Non-cystic	32	Non-cystic	53.1%	Non-cystic	54 (26–81) a	Non-cystic	90 (70–100)	Non-cystic	25% (>5 ​cm)	Non-cystic	N/A	Cystic	N/A
			p-value	N/A	p-value	<0.05	p-value	N/A	p-value	p ​< ​0.05	p-value	N/A	Non-cystic	N/A
													p-value	N/A
													
													pMGMT met	
													Cystic	N/A
													Non-cystic	N/A
													p-value	N/A

a Median age instead of mean age.

b Expressed as mean T1Gd volume.

The average age was 33.4 years for patients with cystic glioblastomas and 58.9 years for those with non-cystic glioblastomas. All seven studies in Table 2 reported that patients with cystic glioblastoma were younger on average than those with non-cystic tumors. Of the seven studies, four (Utsuki et al., 2006; Choi et al., 2010; Curtin et al., 2020; Kaur et al., 2011) showed a significant difference (mean age 52.1 and 58.3 years respectively), and three (Utsuki et al., 2006; Choi et al., 2010; Curtin et al., 2020) of these four studies also reported positive survival outcome in cystic over non-cystic cases. The prognosis of glioblastoma has been reported to be better for younger patients (Ulutin et al., 2006). The presence of a cyst was also found to be associated with a survival advantage when controlling for age (Curtin et al., 2020). Of the three papers that reported pre-operative KPS, only Curtin et al. (2020) showed a higher KPS among cystic compared to non-cystic cases. Although summative data suggests that there are more male (n ​= ​799) than female (n ​= ​532) patients in both cystic and non-cystic glioblastoma cases, none of the studies reported a significant difference between the two groups.

### 3.6 The frontal lobe is the most frequent anatomical location of cystic glioblastomas

Of these 20 studies, three case series (Bink et al., 2005; Chakravarthi et al., 2019; Khanna et al., 2018) and six retrospective studies (Utsuki et al., 2006; Kaur et al., 2011; Jallo et al., 1997; Nakamura et al., 2011; Nakata et al., 2017; Roh et al., 2017) reported the anatomical location of cystic glioblastomas (Supplementary Table 1). The frontal lobe was the most frequently affected (43%), followed by the temporal lobe (28%).

### 3.7 Cystic glioblastomas are larger than non-cystic glioblastomas but grow more slowly

Five reported the average size of tumors and all concluded that cystic tumors are larger than non-cystic tumors, with three out of five papers reporting a significant difference (Table 2). Only one study (Curtin et al., 2020) compared tumor growth velocity on MRI and showed a significant difference (p ​= ​0.039) between cystic and non-cystic glioblastoma with growth velocities of 0.03 ​mm/day and 0.12 ​mm/day respectively.

### 3.8 The prevalence of IDH1 mutations and pMGMT methylation is similar for cystic and non-cystic glioblastomas

Two studies (Curtin et al., 2020; Wu et al., 2015) compared the prevalence of IDH1 mutation and pMGMT methylation in cystic versus non-cystic glioblastoma and found no significant difference. Both papers reported a significant positive survival outcome in cystic over non-cystic glioblastoma.

## 4 Discussion

Cystic components within glioblastomas are relatively common but their significance is not known. Histologic analysis of the tumor cyst lining helps to distinguish between ‘true’ cysts with an endothelial coating and ‘pseudocysts’ with palisading cellular cavity margins but fails to explain their etiology (Utsuki et al., 2006). Cyst fluid may represent necrotic liquefaction of tumor or brain tissue, actively secreted tumor fluid or cerebrospinal fluid trapping – although differences in protein content argue against the latter (Hoelscher et al., 2013). Alternatively, cyst fluid might represent a serum derivative and might act as a nutrient reservoir that sustains tumor growth (Dahlberg et al., 2017). Cyst fluid has been shown to suppress the activation of lymphocytes *in vitro* (Khanna et al., 2018), implying that cystic glioblastomas may have a negative inflammatory component compared to their non-cystic counterparts (Kikuchi and Neuwelt, 1983).

Previous studies investigating the prognostic impact of cystic glioblastoma have reported conflicting results in part this may be due to the small numbers of patients included in the studies and the relative infrequency of cystic compared to non-cystic glioblastomas. In the current study our results demonstrate that cystic glioblastoma is associated with a longer overall survival compared to non-cystic glioblastoma. However, some of the studies included predate the 2016 WHO classification system and did not test IDH status. In addition, several papers (Utsuki et al., 2006; Choi et al., 2010, 2020; Li et al., 2016; Pope et al., 2005) did not specifically mention whether the glioblastoma was new or recurrent, both are potential confounding factors.

In the current study no significant difference in IDH1 mutation status and pMGMT methylation was identified. This supports the notion that the improved survival seen in cystic glioblastoma is most likely not simply due to confounding IDH1 mutation or pMGMT methylation status. None of the papers included in this review compared the presence of 1p/19q co-deletion or IDH2 mutation status. Zhou (Zhou et al., 2018) suggested that the association of cystic glioblastoma with telomere length and pMGMT methylation should be analyzed.

It is possible that sex or other patient related factors are responsible for the survival difference seen in cystic glioblastomas. Curtin et al. (2020) reported that presence of cyst is associated with overall survival benefit in males but not females, and that females who received current SOC with non-cystic glioblastomas showed longer overall survival compared to females with cystic glioblastoma but no explanation for this potential difference was identified. Furthermore, the frontal lobe was most frequently affected by cystic glioblastomas, and a longer OS has been reported for glioblastomas located in frontal and centro-parietal compared to other regions of the brain (Lohle et al., 1992). However, none of the available studies compared glioblastomas of different brain regions.

Cystic tumors are often reported to be larger on average than non-cystic tumors (Utsuki et al., 2006; Choi et al., 2010, 2020). Studies measured tumor size using different methods (surface area measurements or volumetric analysis with T1Gd MRI) limit validity of direct comparison. However, no significant difference in OS was found between those that defined cystic glioblastoma as any tumor cyst (Choi et al., 2010; Curtin et al., 2020; Li et al., 2016; Pope et al., 2005; Yamasaki et al., 2019) and those that included cysts comprising >50% of tumor volume (Maldaun et al., 2004; Utsuki et al., 2006; Kaur et al., 2011; Sarmiento et al., 2014). This may suggest that a cystic component is associated with improved survival regardless of its size. Nevertheless, these differences create a potential selection bias. One study showed that cystic tumors grow at slower rate than non-cystic tumors (Curtin et al., 2020) which might account for an element of improved survival. This is consistent with the observation that cyst fluid is able to suppress of the activation of lymphocytes *in vitro* (Kikuchi and Neuwelt, 1983) implying that cystic glioblastomas may be biologically different than their non-cystic counterparts and the tumor cells may be exposed to a different (potentially suppressive) microenvironment (Dahlberg et al., 2017).

The standardization of care after the introduction of the Stupp protocol (Stupp et al., 2005) may confound the comparison of survival outcomes. We found no significant difference in outcomes between studies from before and after 2005 but it is possible that cystic and non-cystic glioblastomas may respond to the SOC differently. Resection of cyst components, or permanent/intermittent drainage of cyst fluid, has been suggested to reduce tumor recurrence (Kim et al., 1999). Instillation of a radionuclide within a tumor cyst with the aim of decreasing the rate of cyst fluid accumulation has also been described in literature (Taasan et al., 1985).

The overall quality of the evidence available for this study was low. Each study within the analysis had at least 2 risk of bias categories at a high or unclear risk of bias. Future prospective studies looking at survival outcome of cystic versus non-cystic glioblastoma that are well controlled for bias are still needed. These studies should include the most recent WHO molecular classification, detailed control for performance status/comorbidities, treatment modality applied, and formal classification of the tumor cysts to reduce heterogeneity. Well-conducted research looking at molecular/pathological, radiomic, and surgical/treatment specific aspects of cystic glioblastoma would be valuable additions to the literature, as many aspects of this anatomico-pathological glioblastoma subtype remain unexplored.

## 5 Conclusion

This meta-analysis compared cystic and non-cystic glioblastomas. Cystic glioblastomas appear to be associated with longer OS, younger patients and a larger tumor volume but slower growth. No significant difference in the proportion of males or females affected or the pre-operative functional status of patients was detected. The prevalence of IDH1 mutation and pMGMT methylation is similar for cystic and non-cystic glioblastomas. The etiology of cystic components and why they might confer a survival benefit remains to be determined. Future studies comparing treatment effects in a formally defined and rigorously controlled cystic/noncystic glioblastoma population would be clinically valuable.

## Ethics approval and consent to participate

This systematic review and meta-analysis is carried out in accordance to guideline provided by Preferred Reporting Items for Systematic Reviews and Meta-Analyses (PRISMA) 2020 Statement.

## Consent for publication

Not applicable.

## Availability of data and materials

All data generated or analyzed during this study are included in this published article and its supplementary information files.

## Funding

Ciaran Scott Hill is funded by a NIHR Academic Clinical Lectureship, a 10.13039/501100000289CRUK Pioneer Award, a 10.13039/501100000691Academy of Medical Sciences Starter Grant, the ULC BRC, and by a 10.13039/100015873National Brain Appeal Innovation Award.

## Authors' contribution

Morrakot Sae-Huang (Conceptualisation, Methodology, Validation, Formal analysis, Investigation, Writing – Original Draft, Writing – Review & Editing, Project administration); Luke Christopher Smith (Methodology, Validation, Formal analysis, Investigation, Visualisation, Writing – Review & Editing); Inga Usher (Conceptualisation, Writing – Review & Editing, Visualisation); Ciaran Scott Hill (Conceptualisation, Methodology, Validation, Writing – Review & Editing, Supervision, Project administration).

## Declaration of competing interest

The authors declare that they have no known competing financial interests or personal relationships that could have appeared to influence the work reported in this paper.
