# Supplementary material for: Cystic glioblastoma: A systematic review and meta-analysis of characteristics and outcomes
Source: Brain Spine. 2022 Nov 21;2:101692. doi: 10.1016/j.bas.2022.101692 (PMC9808441; doi:10.1016/j.bas.2022.101692)
Supplement: Multimedia component 1 [file mmc1.docx]

| **Supplementary Table 1. Studies which report on anatomical location of cystic GBMs.** | | | | | |  |  |  |  |  |  |  |  |  |  |  |
| --- | --- | --- | --- | --- | --- | --- | --- | --- | --- | --- | --- | --- | --- | --- | --- | --- |
| † Studies did not report on left/ right of anatomical position. | | | |  |  |  |  |  |  |  |  |  |  |  |  |  |
|  |  |  |  |  |  |  |  |  |  |  |  |  |  |  |  |  |
| STUDY | STUDY TYPE | FRONTAL | | FRONTO-TEMPORAL | | TEMPORAL | | PARIETAL | | PARIETAL-OCCIPITAL | | OCCIPITAL | | OTHERS | N/A |  |
|  |  |  |  |  |  |  |  |  |  |  |  |  |  |  |  |  |
|  |  | Left | Right | Left | Right | Left | Right | Left | Right | Left | Right | Left | Right |  |  |  |
| Bink et al., 2005 [26] | Case series | -- | -- | -- | -- | 2 | 2 | -- | 2 | 1 | -- | -- | -- | -- | 1 |  |
| (n=6) |  |  |  |  |  |  |  |  |  |  |  |  |  |  |  |  |
| Chakravarthi et al., 2019 | Case series | -- | -- | -- | -- | -- | -- | -- | -- | -- | 1 | -- | -- | 1 (Thalamus) | -- |  |
| [27] (n=1) |  |  |  |  |  |  |  |  |  |  |  |  |  |  |  |  |
| Jallo et al., 1997 [29] | Retrospective cohort study | 3 | | -- | -- | 1 | | 1 | | -- | -- | -- | -- | -- |  |  |
| (n=5) |  |  |  |  |  |  |  |  |  |  |  |  |  |  |  |  |
| Kaur et al., 2001 [15] | Retrospective cohort study | 15 † | | -- | -- | 10 † | | 7 † | | -- | -- | 2 † | | 2 † | -- |  |
| (n=37) |  |  |  |  |  |  |  |  |  |  |  |  |  |  |  |  |
| Khanna et al., 2018 [28] | Case series | 4 † | | 3 † | | -- | -- | -- | -- | -- | -- | -- | -- | -- | -- |  |
| (n=7) |  |  |  |  |  |  |  |  |  |  |  |  |  |  |  |  |
| Nakamura et al., 2011 | Retrospective cohort study | 1 | -- | -- | -- | -- | -- | 1 | -- | -- | -- | -- | -- | -- | 7 |  |
| [30] (n=9) |  |  |  |  |  |  |  |  |  |  |  |  |  |  |  |  |
| Nakata et al., 2017 [31] | Retrospective cohort study | -- | -- | -- | -- | -- | -- | -- | -- | -- | -- | -- | -- | 2 (Cerebellum) | -- |  |
| (n=2) |  |  |  |  |  |  |  |  |  |  |  |  |  |  |  |  |
| Roh et al., 2017 [32] | Retrospective cohort study | -- | 4 | 3 | 1 | 1 | 2 | 2 | 2 | 1 | -- | -- | -- | -- | -- |  |
| (n=16) |  |  |  |  |  |  |  |  |  |  |  |  |  |  |  |  |
| Utsuki et al., 2006 [7] | Retrospective cohort study | 2 † | | -- | -- | 1 † | | 1 † | | -- | -- | 1 † | | -- | -- |  |
| (n=5) |  |  |  |  |  |  |  |  |  |  |  |  |  |  |  |  |
| TOTAL (n=75) | | 29 | | 7 |  | 19 | | 16 | | 3 |  | 3 | | 5 | 8 |  |
